# Effect of pre-season training phase on anthropometric, hormonal and fitness parameters in young soccer players

**DOI:** 10.1371/journal.pone.0225471

**Published:** 2019-11-25

**Authors:** Fabrizio Perroni, Simona Fittipaldi, Lavinia Falcioni, Lucia Ghizzoni, Paolo Borrione, Mario Vetrano, Riccardo Del Vescovo, Silvia Migliaccio, Laura Guidetti, Carlo Baldari

**Affiliations:** 1 Faculty of Psychology, eCampus University, Novedrate, Como, Italy; 2 IRCCS, SDN, Naples, Italy; 3 Department of Movement, Human and Health Sciences, University of Rome “Foro Italico”, Rome, Italy; 4 Department of Medical Sciences, University of Turin, Turin, Italy; 5 Physical Medicine and Rehabilitation Unit, Sant'Andrea Hospital, "Sapienza" University of Rome, Rome, Italy; 6 “AS Roma” Soccer Team, Rome, Italy; Universidade Federal de Mato Grosso do Sul, BRAZIL

## Abstract

The aims of the study were to investigate 1) the effect of 8 weeks of PSP training on anthropometrics, salivary hormones and fitness parameters in youth soccer players, 2) the correlations between fitness and hormonal parameters, and 3) the impact of the experience of the coach and his methodology of training on these parameters. Weight, height, BMI, pubertal development (PDS), salivary Cortisol (sC), salivary Testosterone (sT), salivary sDHEAS, intermittent tests (VO_2max_), and countermovement jump test (CMJ) modifications of 35 youth soccer players (age: 14±0 yrs; BMI: 20.8±1.8 k/m^2^) from two Italian clubs (“Lupa Frascati” -LF-; “Albalonga” -AL) were analysed. A significant (p<0.05) time by club effect was observed in sC (F_(1,31)_ = 9.7, ES = 1.13), sT (F_(1,31)_ = 4.2, ES = 0.74), CMJ (F_(1,28)_ = 26.5, ES = 1.94), and VO_2max_ (F_(1,28)_ = 8.5, ES = 1.10). Statistical differences (p<0.05) in weight (F_(1,32)_ = 25.5, ES = 0.11), sC (F_(1,31)_ = 32.1, ES = 1.43), sT/sC ratio (F_(1,31)_ = 10.1, ES = 0.97), sDHEAS/sC ratio (F_(1,31)_ = 6.3, ES = 0.70), and VO_2max_ (F_(1,28)_ = 64.3, ES = 1.74) were found within time factor. Between clubs, differences (p<0.05) in sC (F_(1,32)_ = 8.5, ES = 1.17), sT (F_(1,31)_ = 4.2, ES = 0.74), CMJ (F_(1,28)_ = 26.5, ES = 1.50), and VO_2max_ (F_(1,28)_ = 8.5, ES = 1.10) were found. CMJ was inversely correlated with sDHEAS (r = -0.38) before PSP, while Δ of CMJ showed significant correlations with Δ of sC (r = 0.43) and ΔVO_2max_ was inversely correlated with ΔBMI (r = -0.54) and ΔsC (r = -0.37) in all subjects. Considering each single club, ΔVO_2max_ showed correlations with ΔBMI (r = -0.45) in AL, while ΔCMJ showed correlations with ΔPDS (r = 0.72) in LF club. Since the PSP is often limited training time to simultaneously develop physical, technical and tactical qualities, an efficient method to distribute the training load is important in youth soccer players to increase the performance and to avoid injuries.

## Introduction

Soccer is the most popular sport worldwide, particularly among children and adolescents [1]. During a young soccer match, it has been demonstrated [2–3] high internal load in young soccer players and a significant influence of age and playing position on the distances covered and the intensity of running.

Physical fitness of soccer players is usually measured in terms of endurance, speed, power, and strength. Many studies showed a relationship between aerobic power (VO_2max_) and competitive ranking, quality of play, distance and high-intensity activity covered during the match, playing positions and changes throughout the season [4–6], while Counter Movement jump (CMJ) was considered to be functional for optimal performance in soccer and a good predictor of explosive capability for talent identification [7].

It is well known that hormones play a pivotal role in the maintenance of physiological homeostasis and in the optimal responses to stressful physical and/or psychological events. In particular, Dehydroepiandrosterone Sulphate (DHEAS), Testosterone (T) and Cortisol (C) are hormones that play an important role in the daily physiological functions, in particular, when exercise related stress is concerned. As anabolic hormone, T has been shown to be indirectly involved in a number of short-term processes which support healthy muscle development (e.g. regulation of lipid/protein, neural activity and energy metabolism) [8] while C reflects both physical and psychological modification associated with environmental stresses and it is considered to be a strong indicator of catabolic metabolism [9]. Moreover, the T/C ratio has been positively related to changes in physical performance [10]. In contrast, DHEAS is a steroid classified as a weak androgen which possess anti-stress effect [11] and DHEAS/C ratio might be an index of the degree to which an individual is protected against the negative effect of stress [12]. Regarding soccer athletes, Handziski et al. [13] have shown significant differences in hormones (ACTH, cortisol, testosterone and testosterone/cortisol ratio) in adults professional soccer players between three phases of soccer training process of a competition season, while Di Blasio et al. [14] have shown that changes induced by training sessions and matches on the hypothalamic–pituitary–adrenal and hypothalamic–pituitary–testis axes are significantly different, and show different trends through the season.

It has been highlighted [15] that the physiological response to a given training load (TL) is considered an important component of training and that its precise evaluation can give to practitioners an ability to prescribe training with confidence and with a predictable outcome in a defined period. In soccer, the target of periodization is to appropriately manage the TL to optimise the competitive performance. Training in ‘‘pre-season” phase (PSP) usually focuses to obtain a basic level of fitness in players following the ‘‘off-season” period to allow them to run a successful competitive season, during which the focus is the maintenance of the specific capabilities developed during PSP [16]. Despite the competitive season lasts several months (~10) in the soccer sport, the PSP is characterized by the short duration (few weeks) and, generally, by a gradual increase up to 2.4 times in the training loads [17].

Study of Bangsbo et al. [18] highlighted that the weekly training programmes of elite adult soccer players vary according to the phase of the annual plan, the number of training and match during the competitive season (usually more than 100 training sessions and 40 matches), and/or the experience of the coach. Usually, training in young soccer players reflects adult training without considering that the performance capability of a young player is closely related to the maturity status [19]. Thus, aims of the present study were to investigate 1) the effect of 8 weeks of PSP training on anthropometrics (weight, height and Body Mass Index), salivary hormones (C, T, and DHEAS) and fitness (CMJ and VO_2max_) parameters in youth soccer players, 2) the possible correlations between fitness and hormonal parameters, and 3) the impact of the experience of the coach and his methodology of training on these parameters.

To our knowledge, this is the first attempt to evaluate the effect of PSP soccer training on amateur youth soccer players by multifactorial approach.

## Methods

### Experimental design

The aim of the study was to investigate the physiological and performance changes over PSP intra youth soccer players. To permit a better adaptation of TL to increase the performance and to avoid injuries, pre-season soccer camp has been organized in 8 weeks. Each of the 8 weeks of the pre-season soccer camp included six training days and one day of rest with one training session/day the first 4 weeks, and 3 training session/week performed for the following 5–8weeks (120-min daily training units; from 5:00 to 7:00 pm).

The measurement consisted of anthropometric and physical examination, determination of salivary hormones, and pubertal development questionnaire. Measurements were carried out before (pre) and after (post) the PSP:

1. *Pre*: Two day before the training session (August, resting values)
2. *Post*: At the end of 8 weeks of high intensity training programme, at the beginning of the Italian competitive soccer season (in the last week before the first official match; October)

Each testing session was performed in two days with the same sequence (Day 1: hormonal collection, pubertal development, anthropometric, and jumping evaluations; Day 2: aerobic evaluations), at approximately the same time of the day (between 4:00 and 5:00 pm), and is similar environmental conditions (temperature:18–20° C; humidity: 50–60%) to minimize circadian rhythms, nutrition, and climate-related factors. Since appropriate diet and fluid intake enhance training adaptations, during the residential training camp a clubs medical doctor planned athlete’s diet. To reduce the influence of weather conditions, training sessions and experimental evaluations were performed on an artificial turf approved for national level competitions. The Soccer Clubs considered these evaluations as a routine exam of their young soccer players, and they agreed to the participation of their youth soccer players in the study. To reduce measurement variation, the same experienced investigator conducted all the evaluations in the pre and post PSP. Subjects were instructed to avoid food and drink in the hour before testing and to avoid any relevant stressor (physical exercise and/or major stressful activities, etc.) 24 hours before testing session.

Puberty was evaluated by a self-administered rating scale for pubertal development (PDS) questionnaire [20], which was completed in a quiet room upon arrival to the club. After completing the questionnaire, subjects were conducted to the medical room where their anthropometric measurements (weight, height, and body mass index) were taken.

Before starting anaerobic and aerobic tests, players underwent a 15-minute standardized warm-up period consisting of jogging (40–60% of individuals’ theoretical maximal Heart Rate, calculated as 220-age), strolling locomotion, and stretching.

To enhance the positive engagement of participants, players were informed about the aims of the study and verbally encouraged to perform the tests with full concentration and maximum effort.

Considering the nature of the jumping activities and the frequency with which they occur in a typical soccer match, we used the CMJ, according to the protocol described by Bosco et al. [21], to evaluate the anaerobic capacity of youth soccer players. In addition, to estimate the maximal oxygen consumption of subjects, we used the Yo-Yo Intermittent Recovery Test Level 1 [4] that it has been shown to be a reliable, valid measurement of match-related fitness performance in soccer [5].

All procedures performed in the study were approved by the Bioethics Committee of University of Turin (Study Protocol n° 134685) and they were in accordance with the ethical standards of the institutional and/or national research committee and with the 1964 Helsinki declaration and its later amendments or comparable ethical standards.

### Participants

We recruited 45 male young soccer players (age: 165±3 months; BMI: 20.8±1.8 kg/m^2^) who took part in the PSP training phase after 45 days off-season holidays in which they didn’t carry out specific activities such as soccer but only recreational sports fitness. However, ten of the 45 players were excluded from the final analysis due to injuries, or lack of follow-up tests, or >10% of absence at the training sessions. Thus, 35 participants were included in the final analysis. Participants belonged to two different Italian youth clubs (“Lupa Frascati”–LF-: n: 15; and “Albalonga” -AL-: n: 20) of the same geographic area (Rome, Italy), with same chronological age (year of birth: 2003) and competing in the same youth Italian regional category (“Giovanissimi”). Players lived in their own homes with their respective families, and they possessed at least 6 years of competitive and training experience. All subjects were in good health and were not taking medication, nutritional supplements (i.e., amino acids, etc.) or drugs, including anabolic-doping agents that could influence the experimental protocol. Before the study, after verbal and written explanation of the experimental design of the study, a signed consent form was filled by parents of all soccer players (<18 years). In addition, all players were fully accustomed to the procedures used in this research and were informed that they could withdraw from the study at any time.

During the PSP, standardized training sessions were organized keeping constant the duration (120 min) and content of training. The generic training programme and competition plan were developed by the technical clubs’ coaches and were not influenced by the experimental study. Before the beginning of the first training day, to allow a good training plan, researchers informed the coaches of the Clubs about the results of tests. During weeks 1–4, the training focused on physical conditioning aimed to the development of the basic level of aerobic and anaerobic performance, muscle strength training with free weights and/or machine weights and technical and tactical (Te/Ta) skills. During weeks 5–8, the conditioning training was slightly modified: aerobic and anaerobic performance, functional muscle strength training with plyometric drills, sprint training drills and Te/Ta. Postural and stretching exercises, primarily focused on the muscles of the lower body and back, were performed every day at the end of training sessions (5 min).

Methodology of training of each single club was built by their coach based on the personal background and on specific coaching soccer experience (“Table 1”).

**Table 1:** Differences between background, soccer experience and methodology of training of each coach.

	LF	AL
*License*	UEFA B	UEFA B
*year of birth*	1981	1971
*experiences as soccer player*	amateur leagues (10 yrs)	Professional (3yrs), semi-professional (6yrs), amateur leagues (15 yrs)
*experiences as soccer trainer*	“Giovanissimi” (4 yrs)	“Primavera” (1yrs), “Basic activity” (1yrs), “Giovanissimi” (3 yrs)
*percentage of time spent to increase resistance with ball*	40%	80%
*percentage of time spent to increase strength training*	60% explosive methods 40% maximal strength	80% explosive methods 20% maximal strength
*percentage of time spent to increase Volume*	40% (60% Intensity)	40% (60% Intensity)
*percentage of time spent to increase the physical conditioning in a single training unit*	60%	40%
*percentage of time spent to increase the Technical/Tactical component in a single training unit*	40%	60%

None of the participants underwent any strenuous activity and training outside of their normal training schedule.

### Anthropometric evaluation

Weight (kg) and height (m) were measured in light clothes, without shoes, using an electronic scale (±0.1 kg) and a stadiometer (±0.1 cm) (Seca 702, Seca GmbH & Co. KG, Hamburg, Germany). Body Mass Index (BMI) was calculated as weight (kg)/height^2^ (m^2^) and was used to asses weight relative to height.

### Pubertal evaluation

Evaluation of pubertal development (PDS) was obtained by a reliable and valid brief Self-Administered Rating Scale for Pubertal Development questionnaire as previously published [20]. Perroni et al. [22] translated the English version of Carskadon and Acebo [20] and found a good Cronbach’s alpha (0.85) for internal consistency. In further details. the questionnaire required that youth soccer players to answered 5 questions regarding the amount of change or development of different physical characteristics (body and facial hair growth, skin changes, deepening voice, and growth spurt) associated with pubertal maturation using a Likert 4-point scale (i.e., no development = 1; yes, barely = 2; yes, definitely = 3; no development = 4). PDS scores were derived by summing the rating obtained on the five characteristics and then dividing it by five. In addition, on the basis of reported values of body hair growth, facial hair growth, and voice change, Pubertal Status (PS) have been classified in five pubertal status categories: pre-pubertal (no development on any of three characteristic: a combined score of 3); beginning pubertal (a combined score of 4 or 5); mid-pubertal (a combined score of 6, 7 or 8); advanced pubertal (a combined score of 9, 10 or 11); post-pubertal (completed development of all three characteristics: a combined score of 12). The questionnaires were completed individually, although an investigator was present to help if required.

### Explosive power evaluation

To measure the explosive power of lower extremities, youth soccer players performed a vertical jump test that showed a high test-retest stability coefficients (range 0.80–0.98) [23] on an optical acquisition system (Optojump, Microgate, Udine, Italy) which is triggered by the feet of the subject at the instant of taking-off and at contact upon landing (10^-3^s of resolution). Height of jump was calculated in real time by a specific software [24]. From the standing position, researchers asked to the youth soccer players to bend quickly their knees to a 90° angle and, immediately after, to perform a maximal vertical thrust (stretch-shortening cycle). During the jump, subjects had to keep the hands on the hips to avoid any effect of arm-swing and trying to avoid any knee or trunk countermovement. During the phase of flight, subjects had to keep their body vertical, and land with knees fully extended. The highest of three correct jumps (if soccer players failed to adhere to the rigorous protocol, the trial was repeated) was used for further analysis.

### Aerobic evaluation

The Yo-Yo Intermittent Recovery Test Level 1 (YYIRT1) was used to estimate maximal oxygen consumption (VO_2max_) [4]. The test consisted of repeated 20m runs back and forth between the starting, turning, and finishing line at a progressively increased speed controlled by an audio metronome from a calibrated CD player. Subjects had a 10 s active recovery period (decelerating and walking back to the starting line) between each running bout. When a subject was not able to maintain the required speed and failed twice to reach the finishing line in time, the distance covered at that point was considered the test result [5]. VO_2max_ was estimated from the equation [4]:

VO_2max_ (ml/kg/min) = distance covered (m) × 0.0084 (ml/kg/min)/m + 36.4 (ml/kg/min)

YYIRT1showed an intraclass correlation coefficient (ICC) of 0.78 (from 0.61 to 0.89) [25].

The YYIRT1 were performed in groups of 10–12, on a soccer pitch with players wearing soccer cleats.

### Endocrinological evaluation

Youth soccer players were asked to collect the saliva samples using a cotton swab and saliva collecting tube (DRG International Inc. USA) at the same time of the day (afternoon: 4:00–4:30 pm), in order to avoid the effects of the circadian rhythm and variations in food intake. During saliva collection, contamination with food debris was avoided by rinsing the mouth with water and by delaying the collection for 15 minutes after rinsing to prevent sample dilution. Following saliva collection, collecting tubes were centrifuged at 3000 rpm for 15 min (at 4°C), then saliva samples were stored at –20°C until they were assayed. Each saliva samples were analysed in duplicate for salivary Testosterone (sT), salivary Cortisol (sC), and salivary DHEAS (sDHEAS) using commercially available kits (DRG Diagnostics, Marburg, Germany). Each test was performed, according to manufacture protocol. The sensitivities of sT and sC assays are 2.63 pg/ml (range of detection between 0.94–1000 pg/ml) and 0.537 ng/ml (range of detection: 0.537–80 ng/ml), respectively. The lowest detectable concentration of sDHEA-S is 0.045ng/ml (range of detection 0.045–12 ng/ml). Inter- assay coefficient of variation for the measurements of sC, sT and sDHEAS was less than 8, 10 and 10%, respectively. Intra-assay coefficient of variation for sC, sT and sDHEAS was less than 5, 5 and 7%, respectively.

### Statistical analysis

Data are reported as means and standard deviations (mean ± SD) and all calculations were performed using the statistical package for social sciences IBM SPSS (ver.23, SPSS Inc., Chicago, IL, USA). P-values less than 0.05 were considered statistically significant. An a priori power analysis to determine sample size was completed using G*Power 3.1.9.2 software [26]. The Type I alpha (error level) at 5% and a Type II beta (error level) of 20% (or a power of 80%) were set a priori. This analysis showed that for the medium effect size of .25 and an error probability of .05 and a power of .80, the sample size would need to be N = 34. Thus, the target size of the sample recruited for this study was greater than 34 students. A sample of this size will provide the study with the requisite power needed to provide valid results [27]. Before using statistical test procedures, the assumptions of normality and sphericity were verified by Kolmogorov/Smimov and Mauchly test, respectively. An unpaired Student’s *t*-test was used to assess differences between basal anthropometric characteristic. A 2 time (pre-season vs. post-season) x 2 Soccer Clubs (“Lupa Frascati” vs. “Albalonga”) factors, analysis of variance (ANOVA) with repeated measures on time factor was applied to all variables (height, weight, BMI, PDS, CMJ, VO_2max_, sT, sC, and sDHEAS). When significant interaction effects were established, unpaired Student’s *t*-test was used to assess differences between clubs at a given time point and paired *t*-test to compare pre-season vs. post-season values for each soccer Club. Cohen’s effect sizes (ES) regarding pre-training values were calculated to provide meaningful analysis for comparisons from small groups. Values ≤ 0.2, from 0.3 to 0.6, <1.2 and >1.2 were considered trivial, small, moderate and large, respectively. To analyse relationship among variables (height, weight, BMI, PDS, CMJ, VO_2max_, sT, sC, sDHEAS, sT/sC ratio, and sDHEAS/sC ratio) in the pre- condition and among difference (Δ) between pre and post conditions of each variable, Pearson product-moment correlations were used considering all subjects, and within each clubs. According to Hopkins [28], correlation values < 0.1, from 0.1 to 0.3, from 0.3 to 0.5, from 0.5 to 0.7, from 0.7 to 0.9, > 0.9, and 1.0 were considered trivial, small, moderate, large, very large, nearly perfect and perfect, respectively.

Coefficient of determination (r^2^) was used to assess the proportion of the variance in the dependent variable that is predictable from the independent variables.

## Results

Anthropometric and hormonal and fitness characteristics of the subjects enrolled in the study are shown in “Table 2”, while interaction effect between factors is shown in “Table 3”.

**Table 2:** Means, standard deviations (Means ± SD) and range of anthropometric, hormonal, and fitness characteristics of the study sample.

		*"LF" Group (n*: *15)*						*"AL" Group (n*: *20)*						ES (LF vs AL)	*Total (n*: *35)*					
		*Mean*	*±*	*DS*	*Range*		ES (pre vs post)	*Mean*	*±*	*DS*	*Range*		ES (pre vs post)		*Mean*	*±*	*DS*	*Range*		ES (pre vs post)
					*Min*	*Max*					*Min*	*Max*						*Min*	*Max*	
***Weight (kg)***	Pre	56.81	±	9.72	*42*.*00*	*76*.*90*	0.14	61.95	±	7.97	*48*.*10*	*76*.*40*	0.09	0.58	60.09	±	8.89	*42*.*00*	*76*.*90*	0.11
	Post	58.17	±	10.18	*42*.*20*	*77*.*80*		62.72	±	7.76	*49*.*90*	*77*.*00*		0.50	60.84*	±	8.98	*42*.*20*	*77*.*80*	
***Heigth (m)***	Pre	166	±	6	*158*	*181*	0.18	171	±	8	*157*	*189*	0.13	0.70	169	±	7	*157*	*2*	0.13
	Post	167		5	*158*	*181*		172	±	8	*157*	*190*		0.74	170	±	8	*158*	*2*	
***BMI (m/kg***^***2***^***)***	Pre	20.36	±	2.05	*16*.*82*	*23*.*81*	0.16	21.03	±	1.66	*17*.*88*	*23*.*84*	0.03	0.36	20.75	±	1.81	*16*.*82*	*23*.*84*	0.09
	Post	20.73	±	2.50	*16*.*67*	*24*.*72*		21.08	±	1.80	*18*.*03*	*24*.*30*		0.16	20.94	±	2.09	*16*.*67*	*24*.*72*	
***PDS (UA)***	Pre	2.40	±	0.56	*1*.*20*	*3*.*40*	0.35	2.54	±	0.51	*1*.*40*	*3*.*40*	0.04	0.26	2.48	±	0.52	*1*.*20*	*3*.*40*	0.14
	Post	5.59	±	0.52	*1*.*60*	*3*.*40*		2.52	±	0.43	*1*.*80*	*3*.*40*		0.15	2.55	±	0.46	*1*.*60*	*3*.*40*	
***sT (ng/ml)***	Pre	0.07	±	0.04	*0*.*02*	*0*.*15*	0.17	0.08	±	0.03	*0*.*01*	*0*.*14*	1.33	0.28	0.07	±	0.04	*0*.*01*	*0*.*15*	0.22
	Post	0.08	±	0.07	*0*.*02*	*0*.*24*		0.04*#	±	0.03	*0*.*01*	*0*.*10*		0.74	0.06	±	0.05	*0*.*01*	*0*.*24*	
***sC (ng/ml)***	Pre	3.31	±	2.60	*0*.*68*	*10*.*65*	1.00	7.75#	±	4.71	*2*.*30*	*15*.*40*	1.96	1.17	5.85	±	4.49	*0*.*68*	*15*.*40*	1.43
	Post	1.42*	±	0.59	*0*.*70*	*2*.*77*		1.16*	±	0.71	*0*.*38*	*2*.*69*		0.40	1.27*	±	0.66	*0*.*38*	*2*.*77*	
***DHEAS (ng/ml)***	Pre	3.64	±	2.51	*1*.*08*	*10*.*95*	0.27	3.34	±	2.63	*0*.*50*	*11*.*48*	0.02	0.12	3.48	±	2.54	*0*.*50*	*11*.*48*	0.12
	Post	2.93	±	2.66	*1*.*01*	*10*.*86*		3.27	±	3.40	*0*.*65*	*12*.*79*		0.11	3.12	±	3.06	*0*.*65*	*12*.*79*	
***sT/sC (ng/ml)***	Pre	0.03	±	0.04	*0*.*004*	*0*.*13*	0.58	0.01	±	0.01	*0*.*002*	*0*.*04*	1.37	0.68	0.02	±	0.03	*0*.*00*	*0*.*13*	0.97
	Post	0.06	±	0.06	*0*.*01*	*0*.*21*		0.05	±	0.04	*0*.*01*	*0*.*14*		0.20	0.06*	±	0.05	*0*.*01*	*0*.*21*	
***DHEAS/sc (ng/ml)***	Pre	1.42	±	0.82	*0*.*25*	*3*.*03*	0.57	0.54	±	0.40	*0*.*00*	*1*.*56*	0.86	1.36	0.94	±	0.76	*0*.*00*	*3*.*03*	0.70
	Post	2.25	±	1.89	*0*.*52*	*7*.*29*		4.63	±	6.70	*0*.*61*	*28*.*65*		0.48	3.58*	±	5.24	*0*.*52*	*28*.*65*	
***Cmj (cm)***	Pre	25.23	±	2.57	*21*.*50*	*33*.*90*	1.62	30.47#	±	4.26	*23*.*80*	*38*.*30*	0.66	1.50	28.51	±	4.49	*21*.*50*	*38*.*30*	0.01
	Post	29.40*	±	2.58	*25*.*80*	*32*.*30*		28.04*	±	2.97	*21*.*40*	*32*.*00*		0.49	28.54	±	2.86	*21*.*40*	*32*.*30*	
***VO***_***2max***_ ***(ml/kg/min)***	Pre	42.93	±	1.23	*40*.*10*	*46*.*14*	0.62	42.92	±	1.81	*39*.*93*	*44*.*13*	2.20	0.01	42.92	±	1.59	*39*.*93*	*46*.*14*	1.74
	Post	44.95*	±	2.08	*41*.*44*	*48*.*50*		47.25*#	±	2.11	*43*.*10*	*50*.*50*		1.10	46.41*	±	2.35	*41*.*44*	*50*.*50*	

*LF* = Lupa Frascati; *AL* = Albalonga; *Total* = all subjects

*Significant differences vs pre

# Significant differences vs LF group

**Table 3:** Interaction effect of all variables between factors.

Variable	Factors	F	df	P	Partial ɳ^2^
*Weight (kg)*	season by club	2.022	1	0.16	0.059
	season	25.5	1	<0.001	0.444
	club	2.543	1	0.121	0.074
*Heigth (m)*	season by club	0.11	1	0.743	0.003
	season	2.201	1	0.148	0.064
	club	4.097	1	0.051	0.114
*BMI (m/kg*^*2*^*)*	season by club	1.654	1	0.208	0.049
	season	3.108	1	0.087	0.89
	club	0.567	1	0.457	0.017
*PDS (UA)*	season by club	0.875	1	0.18	0.55
	season	1.216	1	0.278	0.037
	club	0.056	1	0.815	0.002
*sT (ng/ml)*	season by club	4.215	1	0.049	0.12
	season	0.887	1	0.354	0.028
	club	2.854	1	0.101	0.084
*sC (ng/ml)*	season by club	9.749	1	0.004	0.239
	season	32.13	1	<0.001	0.509
	club	8.55	1	0.006	0.216
*DHEAS (ng/ml)*	season by club	0.015	1	0.905	<0.001
	season	0.823	1	0.371	0.026
	club	0.129	1	0.722	0.004
*sT/sC (ng/ml)*	season by club	0.055	1	0.815	0.002
	season	10.105	1	0.003	0.246
	club	3.579	1	0.068	0.104
*DHEAS/sC (ng/ml)*	season by club	2.393	1	0.132	0.072
	season	6.272	1	0.018	0.168
	club	0.306	1	0.584	0.01
*CMJ (cm)*	season by club	24.466	1	<0.001	0.489
	season	1.826	1	0.187	0.061
	club	3.225	1	0.083	0.013
*VO*_*2max*_ *(ml/kg/min)*	season by club	8.557	1	0.007	0.234
	season	64.272	1	<0.001	0.697
	club	3.762	1	0.063	0.118

Interestingly, significant time by clubs interaction effect was observed in sC (F_(1,31)_ = 9.7, p<0.01, ɳ^2^ = 0.24), sT (F_(1,31)_ = 4.2, p<0.05, ɳ^2^ = 0.12), CMJ (F_(1,28)_ = 26.5, p<0.01, ɳ^2^ = 0.49), and VO_2max_ (F_(1,28)_ = 8.5, p<0.01, ɳ^2^ = 0.23), while within time factor results demonstrate statistical differences (p<0.05) in weight (F_(1,32)_ = 25.5, p<0.01, ɳ^2^ = 0.44), sC (F_(1,31)_ = 32.1, p<0.01, ɳ^2^ = 0.51), sT/sC ratio (F_(1,31)_ = 10.1, p<0.01, ɳ^2^ = 0.25), sDHEAS/sC ratio (F_(1,31)_ = 6.3, p<0.05, ɳ^2^ = 0.17), and VO_2max_ (F_(1,28)_ = 64.4, p<0.01, ɳ^2^ = 0.70).

As expected, no differences in the anthropometric and pubertal characteristics were found between the young soccer players of the two different Clubs. Considering the total population of subjects, high percentage were in mid-pubertal status (pre: 60%; post: 54%).

Correlations between anthropometrics, PDS, and hormonal variables for basal values and Δ (post-pre) values are presented in “Table 4” and “Table 5”.

**Table 4:** Correlation among anthropometrics (age. weight. height. BMI). Pubertal development Scale (PDS). and hormonal (sC. sT. sDHEAS. sT/sC ratio and sDHEAS/sC ratio) parameters before the “pre-season” evaluation in all subjects and in a single team (LF and AL).

Category	Variables	sC	sT	DHEAS	sT/sC ratio	sDHEAS/sC ratio	Weight	Height	BMI	PDS
*LF*	**sC**	1								
	**sT**	0.04	1							
	**DHEAS**	0.34	-0.19	1						
	**sT/sC ratio**	-0.75**	-0.55*	-0.39	1					
	**sDHEAS/sC ratio**	-0.68**	-0.17	0.41	0.40	1				
	**Weight**	0.39	-0.28	0.18	-0.49	-0.11	1			
	**Height**	0.48	-0.23	0.14	-0.58*	-0.31	0.91**	1		
	**BMI**	0.28	-0.30	0.27	-0.48	0.002	0.95**	0.79**	1	
	**PDS**	0.33	-0.9	0.40	-0.29	0.01	0.63*	0.49	0.69**	1
*AL*	**sC**	1								
	**sT**	-0.17	1							
	**DHEAS**	0.32	0.30	1						
	**sT/sC ratio**	-0.84	0.63**	-0.14	1					
	**sDHEAS/sC ratio**	-0.610**	0.37	0.52*	0.62**	1				
	**Weight**	0.06	0.04	0.16	0.03	0.21	1			
	**Height**	0.06	0.11	0.90	0.01	0.09	0.78**	1		
	**BMI**	-0.002	-0.08	0.03	0.09	0.17	0.68**	0.13	1	
	**PDS**	-0.34	0.44	-0.39	0.51*	-0.003	0.17	0.34	0.03	1
*All subjects*	**sC**	1								
	**sT**	0.63	1							
	**DHEAS**	0.19	0.26	1						
	**sT/sC ratio**	-0.78**	0.53**	-0.20	1					
	**sDHEAS/sC ratio**	-0.751**	-0.01	0.45**	0.59**	1				
	**Weight**	0.21	-0.15	0.13	-0.27	-0.04	1			
	**Height**	0.29	-0.04	0.88	-0.26	-0.16	0.87**	1		
	**BMI**	0.08	-0.19	0.10	-0.18	0.02	0.81**	0.46*	1	
	**PDS**	0.09	0.19	0.07	0.72	-0.03	0.42**	0.42*	0.36*	1

**p< 0.01

*p< 0.05

*Note*: LF = Lupa Frascati Team

AL = Albalonga Team

BMI = Body Mass Index

PDS = pubertal development scale

sC = salivary Cortisol

sT = salivary Testosterone

**Table 5:** Correlation among anthropometrics (age. weight. height. BMI). Pubertal development Scale (PDS). and hormonal (sC. sT. sDHEAS. sT/sC ratio and sDHEAS/sC ratio) parameters in Δ of all subjects and of a single team (LF and AL).

Category	Variables	ΔsC	ΔsT	ΔDHEAS	ΔsT/sC ratio	ΔsDHEAS/sC ratio	ΔWeight	ΔHeight	ΔBMI	ΔPDS
*LF*	**ΔsC**	1								
	**ΔsT**	-0.75	1							
	**ΔDHEAS**	0.52*	-0.16	1						
	**ΔsT/sC ratio**	-0.47	0.54**	-0.24	1					
	**ΔsDHEAS/sC ratio**	-0.31	-0.19	0.49	0.04	1				
	**Δweight**	-0.02	-0.01	0.08	-0.01	0.08	1			
	**ΔHeight**	0.16	0.23	-0.07	-0.04	-0.22	-0.13	1		
	**ΔBMI**	-0.20	-0.04	0.06	0.14	0.23	0.67*	-0.76**	1	
	**ΔPDS**	0.58*	-0.06	0.29	-0.35	-0.45	0.05	-0.13	-0.16	1
*AL*	**ΔsC**	1								
	**ΔsT**	-0.05	1							
	**ΔDHEAS**	0.44	0.38	1						
	**ΔsT/sC ratio**	-0.56*	0.12	0.21	1					
	**ΔsDHEAS/sC ratio**	-0.32	0.01	0.35	0.84**	1				
	**Weight**	0.22	0.15	0.17	-0.18	-0.26	1			
	**ΔHeight**	-0.08	-0.12	-0.27	-0.04	-0.18	0.19	1		
	**ΔBMI**	0.23	0.46**	0.37	-0.17	-0.16	0.54*	-0.53*	1	
	**ΔPDS**	-0.21	0.18	-0.19	0.003	-0.17	0.05	0.57**	-0.18	1
*All subjects*	**ΔsC**	1								
	**ΔsT**	0.93	1							
	**ΔDHEAS**	0.41*	0.09	1						
	**ΔsT/sC ratio**	-0.44**	0.50**	-0.04	1					
	**ΔsDHEAS/sC ratio**	-0.44**	-0.16	0.40*	0.39*	1				
	**ΔWeight**	0.25	0.13	0.22	-0.11	-018	1			
	**ΔHeight**	-0.09	-0.001	-0.19	-0.05	-0.11	0.46	1		
	**ΔBMI**	0.19	0.22	0.21	-0.11	-0.12	0.60**	-0.66**	1	
	**ΔPDS**	0.17	0.11	0.05	-0.12	-0.29	0.11	0.36	-0.09	1

**p< 0.01

*p< 0.05

*Note*: LF = Lupa Frascati Team

AL = Albalonga Team

BMI = Body Mass Index

PDS = pubertal development scale

sC = salivary Cortisol

sT = salivary Testosterone

In addition, considering fitness parameters, CMJ showed significant relationship only with sDHEAS (r = -0.38, r^2^ = 0.14, p<0.05) before the PSP, while Δ of CMJ showed significant correlations with Δ of sC (r = 0.43, r^2^ = 0.18, p<0.05) and Δ of VO_2max_ showed significant correlations with Δ of BMI (r = -0.54, r^2^ = 0.29, p<0.01) and Δ of sC (r = -0.37 r^2^ = 0.12, p<0.01) in all subjects. Finally, considering each single club, Δ of VO_2max_ showed significant correlations only with Δ of BMI (r = -0.45, r^2^ = 0.20, p<0.05) in AL club, while Δ of CMJ showed significant correlations with Δ of PDS (r = 0.72 r^2^ = 0.51, p<0.01) in LF club.

## Discussion

The main findings of this study were 1) significant differences within time (pre vs. post) and between clubs in anthropometric, hormonal and fitness parameters, 2) significant statistical correlations between fitness and hormonal parameters.

Previous studies performed to evaluate potential relationship between anthropometric characteristics and performance in young soccer players [29, 30] demonstrated that anthropometric values are pivotal factors for the success of soccer players. The present study demonstrates similar outcomes regarding height and weight (weight: 60.68 ± 8.38 kg; height: 1.69 ± 0.07 m) to the results previously published by our group [31] in which we recognized anthropometric and somatotype differences among different playing positions within official federation categories in Italian young soccer players. In comparison to the study of Markovic and Mikulic [32] on young male soccer players (from U13 to U19) of a successful club competing in Croatia’s first soccer league, all our soccer players were higher and heavier when compared to their U15 in both pre and post PSP. The increase in body weight at the end of PSP of our soccer players could be the result of a decrease in percent body fat and an increase of muscle mass due at the high training volume of this phase, composed by prolonged endurance training and strength training, and friendly games that use fat stores and causes a large energy expenditure [33].

Iuliano-Burns et al. [34] have previously shown a large range of variability among individuals of the same chronological age in somatic and biological growth, especially accentuated during the adolescent growth spurt, young sport competitions children are divided according to their chronological age. Study of Di Luigi et al. [35] observed the presence of high inter–individual variability of different biological parameters within athletes selected by chronological age in the same class and the presence of significant correlations between chronological age, puberty and the steroid hormone responses to physical exercise. Moreover, previous study of our group [36] demonstrated significant correlation between PDS and CMJ within Italian “Giovanissimi” category. As in this study, we have found the higher frequency distributions of subjects enrolled in the study in mid-pubertal status (55%). Considering that the variation of physiological and fitness variables (i.e. mobility, aerobic and anaerobic capabilities, explosive strength) parameters during the growth could influence the performance, assessment of PDS pubertal development in youth soccer categories is very important in order to determine adequate physical training programs according to individual biological characteristics. An efficient method to distribute the TL of these capabilities is important in youth soccer players to avoid injuries.

The results obtained in the present study demonstrated that at the end of the pre-season training phase, C levels decreased in both clubs while T decreased only in AL club. In all subjects we observed an increase in the T/C ratio compared to baseline value. As previously indicated [37], the decrease in the T/C ratio is a reliable index of chronic physical stress due to high intensity and duration of exercise. Interestingly, our results show an increase in T/C values ​​for all athletes as well as for individual clubs, strongly indicating a good training setting. The ability of body to recover and regenerate following composite stresses (i.e. training and competition) can significantly influence physical performance. In fact, the dynamic homeostatic balance between anabolic (building) and catabolic (breakdown) processes within the muscle can influence the quality of a player's performance during training and competition season [29]. Adaptive responses to the stresses associated with training (i.e. physiological and psychological) are indicated by alterations in specific anabolic and catabolic hormones concentrations [38]. Compared to basal level, significantly elevated levels of both C and T have been demonstrated during a competitive soccer load season [39]. Results of our groups [35] confirmed significant improvement of sC (Pre: 19.76 nmol^.^L^–1^ vs post: 22.56 nmol^.^L^–1^) in acute response to soccer exercise in young soccer player. In contrast, Hammami et al. [40] showed significant alterations in both hormonal concentrations and physical fitness parameters (jumping tests and 30m sprint) in the young soccer players belonging to the U17 Tunisian national clubs during the two seasons follow-up. Baldari et al. [41] showed a positive correlation (r = 0.38, r^2^ = 0.14) between salivary DHEAS concentrations and explosive leg power at the beginning of competitive soccer season, while Gravina et al. [42] reported a positive correlation between ΔsT and the improvement in performance in the CMJ (r = 0.48, r^2^ = 0.23) and VO_2max_ (r = 0.32, r^2^ = 0.10) in young soccer players at the end of the season (7 mounts). In our study, after 8 weeks of PSP, the ΔsC was the only hormonal parameter correlated to fitness changes with the coefficients of determination explaining the 12% and 18% of the variance of the ΔVO_2max_ and ΔCMJ (r^2^ = 0.12 and r^2^ = 0.18 respectively). Thus, we suppose that the differences in pre and post PSP represent a chronic adaptation to the TL assigned by soccer training. Interestingly, several studies [43, 44] used T and C to determine the psycho-physiological stress/effort induced by a soccer competition. More specifically, T and C showed to be differentially modulated in response to metabolic stress, and their ratio has been reported as an indicator of anabolic/ catabolic activity and used, in sports physiology, as a marker of either, or both, overreaching and overtraining syndromes. However, training programs and trainer’s approach (technical and psychological) have to based more on individual biological evaluations.

As a typical intermittent-type sport, soccer is composed by many and various explosive ballistic movement (i.e. sprinting, kicking, jumping, accelerations and decelerations, tackling and changes of direction) [18] that are characterized by the stretch-shortening cycle (SSC) that develops during the transition from a rapid eccentric muscle contraction to a rapid concentric muscle contraction (acceleration or positive phase) [45]. Despite the time spent performing these explosive movements represent only a small percentage of a match’s total time, these actions discriminate between a successful and an unsuccessful performance and, due to these reasons, players should be trained with an optimal training routine [30]. The results of this study depict significant differences (p<0.01) in CMJ for time by club analysis with values that are higher than the values previously obtained (2017) in the both test conditions and, in addition, in both clubs. Wisloff et al. [46] highlighted an improvement of vertical jump performance in professional soccer players when body weight and plyometric training was frequently performed during the preseason phase. Our results demonstrate that, despite AL trained high percentage of power strength training compared to LF, LF showed a better improvement (AL = -8%: LF = +17%). This result could be due to a stagnation in power performance and low disposal of TL by AL soccer players. Considering that many studies [47, 48] showed contraindications for the use of high-impact plyometrics training with adolescent (i.e. high inherent risk of injury to bones’ growth plates, association with muscle and tendon damage, and marked inflammatory response) adequate choice of administration and monitoring of exercise and TL should be evaluated.

Finally, one of the factors that predispose toward a successful professional soccer career is the endurance capacity, particularly intermittent specific endurance [6]. A study of Markovic and Mikulic [32] indicate that the YYIR1 test distinguishes differences among varying age categories and among various playing positions in youth soccer players. As compared with results of their study, we have found lower values in pre (755 m) and similar values in post (1170 m) PSP in all subject. The increase in aerobic fitness (VO_2max_) in our study is lower (8.1% vs 37%) than previously reported by Dragijsky et al. [49] in a study performed on trained young Czech soccer players (U13) following the PSP, but higher than the improvement (4.5%) found by Kalapotharakos et al. [50] in elite players. These differences could be due to the little percentage of time spent to increase aerobic capability without ball as declared by both our trainers. Considering the key role of hormonal status in determining physical performance (Yo-Yo test and vertical jump test) in pre-adolescent soccer players [51] and the significant relationships between aerobic power and competitive ranking, club level, and total distance covered during the game [33], high attention to administration of TL. The choice of methodology of training during the PSP should be considered a priority by coach which may induce differences in fitness components and performance capabilities in each young soccer club. Although scientific and multidimensional approaches for talent selection have been suggested for some time, most clubs still rely solely on inherently subjective data from coach assessments (the coaches’ eye).

Literature regarding seasonal variation in physiological and physical fitness among soccer players [42, 49–50] demonstrates significant changes (improvement) in PSP with no further significant changes (in some cases decreases) during the competitive season. The findings of our study demonstrate significant differences within time (pre vs. post) and between clubs in anthropometric, hormonal and fitness parameters, which are result of the different soccer experiences of Coach related to training. However, there are limited available data addressing the selection and training of talent and the conflict of interest between the youth team’s long-term preparation, and the need for short-term club results. This issue highlights the necessity of investments in educational programs for coaches and physical trainers that allow to increase the knowledge of the physiological changes, and their evaluation, in youth soccer players to decrease the injuries risks.

Understandably, it was subject to several limitations. First, the lack of control group to compare the maturation process which may interfere with the results. Second, the lack of monitoring of TL of youth soccer players. Third, the use of salivary hormones levels rather than plasma content. It should be emphasized that salivary hormones measurement is a widely used and accepted method to quantify hormones in young players [52] as it avoids stress due to the needle use, moreover, salivary steroid levels reflect the circulating level of free steroid [53]. Furthermore, we avoided as much as possible the effects of circadian rhythm of adrenal secretion by testing all the subjects at the same time of the day and selected subject of the same age but we could not avoid individual variability in hormones secretion. Another limitation concern variability in the hormone’s levels in some samples. Other authors indicate a large variability in magnitude changes of salivary parameters in soccer players [14, 54]. The high testosterone standard deviation in some groups is probably due to the individual variability and/or mood as previously described [52, 55] in male adolescents. Moreover, aggressiveness typical of competitive sports such as soccer can modulate hypothalamic-pituitary-adrenal axis with an activation that could be differentially dependent by individual physiology [14].

In conclusion, considering that in a sport such as soccer the competitive season lasts several months (~10) and the carrier (amateur and/or professional) lasts several years, further studies are highly recommended to better establish and characterize the effects, both at short and at long time, of different soccer experiences of coach on the performance and, most importantly, on the adequate growth and development of young soccer players.

## Conclusion

Considering that, to adapt to training, youth soccer players need adequate stimuli which allow a recovery useful to be ready at the demands of the following training session, coach have to understand the physiological and psychological responses of youth soccer players to each training stimulus and to change it on the basis of the individual responses to training to maximize positive adaptations, avoiding overtraining and injury.

To achieve success in adult sport competition, young soccer player should have talent which it should be trained by the best coaching and training during each step of their development processes. In addition, adequate administration of TL during soccer training is necessary for healthy growth and development of subject. Since the PSP is often limited training time to simultaneously develop physical, technical and tactical qualities with multiple sessions of training often performed in the same day and within 24 hours of one, an efficient method to distribute the TL of these capabilities is important in youth soccer players. The variability for different biological parameters in youth soccer player within the same chronological age is probably too often underestimated in the soccer world. Starting from this observation, we have to stimulate a new trainer’s approach (physical, technical and psychological) based more on individual biological characteristics and sensible phase of development. This would be helpful to train young soccer players to optimize the performance with adequately developing of their physical and psychological characteristics, and to reduce both the drop-out rate and possible exercise-related health and social problems. This should also stimulate educational programs for coaches, athletes and their parents to reduce all environmental factors conditioning sport participation in adolescence.
